# Sugarcane Root Transcriptome Analysis Revealed the Role of Plant Hormones in the Colonization of an Endophytic Diazotroph

**DOI:** 10.3389/fmicb.2022.924283

**Published:** 2022-06-24

**Authors:** Qian Nong, Mukesh Kumar Malviya, Manoj Kumar Solanki, Anjali Chandrol Solanki, Li Lin, Jinlan Xie, Zhanghong Mo, Zeping Wang, Xiu-Peng Song, Xin Huang, Shalini Rai, Changning Li, Yang-Rui Li

**Affiliations:** ^1^Key Laboratory of Sugarcane Biotechnology and Genetic Improvement (Guangxi), Ministry of Agriculture and Rural Affairs, Sugarcane Research Center, Chinese Academy of Agricultural Sciences, Guangxi Key Laboratory of Sugarcane Genetic Improvement, Sugarcane Research Institute, Guangxi Academy of Agricultural Sciences, Nanning, China; ^2^Plant Protection Research Institute, Guangxi Academy of Agricultural Sciences, Nanning, China; ^3^Plant Cytogenetics and Molecular Biology Group, Institute of Biology, Biotechnology and Environmental Protection, Faculty of Natural Sciences, University of Silesia in Katowice, Katowice, Poland; ^4^Department of Agriculture Science, Mansarovar Global University, Bhopal, India; ^5^Department of Biotechnology, Society of Higher Education and Practical Application (SHEPA), Varanasi, India

**Keywords:** sugarcane root, physiological function, gene expression, endophytic diazotroph, inoculation

## Abstract

Some sugarcane germplasms can absorb higher amounts of nitrogen *via* atmospheric nitrogen fixation through the bacterial diazotrophs. Most endophytic diazotrophs usually penetrate through the root, colonize inside the plant, and fix the nitrogen. To assess the plant’s bacterial association during root colonization, strain GXS16 was tagged with a plasmid-bear green fluorescent protein (GFP) gene. The results demonstrated that the strain can colonize roots all the way to the maturation zone. The strain GXS16 showed maximum nitrogenase enzyme activity at pH 8 and 30°C, and nitrogenase activity is less affected by different carbon sources. Further, strain GXS16 colonization response was investigated through plant hormones analysis and RNAseq. The results showed that the bacterial colonization gradually increased with time, and the H_2_O_2_ and malondialdehyde (MDA) content significantly increased at 1 day after inoculation. There were no substantial changes noticed in proline content, and the ethylene content was detected initially, but it decreased with time. The abscisic acid (ABA) content showed significant increases of 91.9, 43.9, and 18.7%, but conversely, the gibberellin (GA_3_) content decreased by 12.9, 28.5, and 45.2% at 1, 3, and 5 days after inoculation, respectively. The GXS16 inoculation significantly increased the activities of catalase (CAT), superoxide dismutase (SOD), polyphenol oxidase (PPO), ascorbate peroxidase (APX), and glutathione reductase (GR) at different timepoint. In contrast, the peroxisome (POD) activity had no changes detected during the treatment. In the case of RNAseq analysis, 2437, 6678, and 4568 differentially expressed genes (DEGs) were identified from 1, 3, and 5 days inoculated root samples, and 601 DEGs were shared in all samples. The number or the expression diversity of DEGs related to ethylene was much higher than that of ABA or GA, which indicated the critical role of ethylene in regulating the sugarcane roots response to GXS16 inoculation.

## Introduction

Sugarcane (*Saccharum officinarum*) is the primary sugar crop in China, but unbalanced amounts of nitrogen fertilizer have been used in its production that widely enhance the ecological toxicity. Typically, 450–750 kg of nitrogen are applied per hectare, more than twice the world average. It is also one of the reasons for the high costs of production (Carvalho et al., 2011; Yang et al., 2014). Some sugarcane germplasms can obtain high levels of nitrogen *via* nitrogen fixation by associated nitrogen fixing bacteria (NFB) (Reinhold-Hurek and Hurek, 2011). Unlike symbiotic nitrogen fixation, the associated NFB in sugarcane are loosely combined with the plant. After the interaction, they have no observable phenomenon and do not form characteristic symbiotic structures, such as root nodules. However, they are highly efficient at fixing nitrogen. According to studies, biological nitrogen fixation, which is fixed by related NFB, can provide 30–60% of the nitrogen for sugarcane growth (Urquiaga et al., 2012). Inoculation with the endophytic NFB may enable sugarcane to benefit from biological nitrogen fixation, thus, reducing planting costs by limiting the application of nitrogen. Currently, in the study of sugarcane nitrogen fixation, the primary methods for inoculating sugarcane with NFB involve soaking sugarcane seed stems with a bacterial solution, pouring the bacterial solution on stubble cane, and co-culturing the sugarcane tissue culture seedlings with NFB during the rooting stage. When these methods are used for inoculation, the NFB primarily enters the plants from the lateral root origin, root tip, root hair, detached root cap, and other tissues. The bacteria promote plant growth by fixing nitrogen, secreting auxin and ferritin, and dissolving phosphorus and potassium, thus, increasing sugarcane yields (Boller and He, 2009; Wei et al., 2014a; Malviya et al., 2019).

Research has shown that plants gradually form an immune system, thereby defensive them from infection by pathogens (Boller and He, 2009; Takken and Tameling, 2009). However, the interaction pattern between endophytic bacteria and the host differs from pathogenic microorganisms. The immune response of the host will not be over-reactive during the process of colonization, thus, avoiding their mistaken eradication by the host (Vandenkoornhuyse et al., 2015). Reactive oxygen species (ROS) in plant cells (Han et al., 2015; Zhou et al., 2015), antioxidant protection systems (Alqueres et al., 2013), and plant hormones (Vargas et al., 2014; Liu et al., 2017) play essential roles during these types of interactions. RNA-seq is a deep-sequencing method that provides the transcriptome profile of model and non-model plants (Haider et al., 2017). RNA-seq technique helps examine gene functions and expression at the transcriptional level and elucidates the molecular mechanisms of specific biological processes (Stark et al., 2019; Simoneau et al., 2021). Transcriptomics technology mainly was utilized to investigate the different functions such as the interaction between microbes and plants in response to water deficiencies, root exudates, salt stress, biotic and abiotic stresses (Chauhan et al., 2019; Khan et al., 2020; Malviya et al., 2020; Moradi et al., 2021). However, using RNA-seq technology, there is minimal evidence on the physiological and molecular interaction mechanism between diazotrophs and sugarcane.

Current studies on endophytic NFB in sugarcane are primarily focused on the following two aspects: first, the isolation of NFB. Sugarcane is inoculated with NFB to confirm their endogenous and growth-promoting effects and measure the efficiency of nitrogen fixation (Wei et al., 2014b; Malviya et al., 2019). Secondly, the regulatory mechanism of the expression of nitrogen-fixing genes is studied using multi-omics technology, and the effects of inoculation with endophytic NFB on the expression of genes or resistance to adversity stress of sugarcane are analyzed (Lery et al., 2010; Vargas et al., 2014; Aguiar et al., 2018; Wang et al., 2019). The root system of sugarcane plays an essential role in nitrogen fixation by endophytic bacteria. However, the physiological and molecular responses of sugarcane roots to the inoculation of endophytic NFB have not been systematically studied. In this study, sugarcane tissue culture seedlings were inoculated with the *Burkholderia* endophytic diazotrop strain GXS16 (Nong et al., 2021), which has a stronger association with nitrogen fixation capacity at the rooting stage, to detect the physiological and biochemical effects and changes in gene expression of the sugarcane roots at various time interval. The focus was on analyzing the impact of diazotroph inoculation on plant hormone-related gene expression. It will explain the recognition and response mechanism of sugarcane roots to GXS16 inoculation and provide a theoretical basis to understand better how NFB promotes sugarcane growth.

## Materials and Methods

### Experimental Materials

*Burkholderia* sp. strain GXS16 was previously isolated from the sugarcane variety Guitang 31. Our previous study reported that strain GXS16 has nitrogenase activity of 2.42 μmol C_2_H_4_ h^–1^ml^–1^, strong ACC deaminase activity, and the ability to secrete auxin and degrade inorganic phosphorus, among others (Nong et al., 2021). The height of the sugarcane plant inoculated with GXS16 increases by more than 15% compared with the control, and its dry weight increases by more than 20%. The efficiency is significantly higher than that of model strain *Gluconacetobacter diazotrophicus* PAL5 (Nong et al., 2021). The plasmid standard of the 16S rRNA gene of GXS16 was prepared and stored. The Sugarcane Research Institute of the Guangxi Academy of Agricultural Sciences (Nanning, China), provided the tissue culture seedlings of sugarcane variety RB86-7515.

### Green Fluorescent Protein Technique for Tagging of Strain

Plasmid transformation has been done as described by Malviya et al. (2019). *Burkholderia* strain was chosen as a recipient for genetic tagging with GFP-pPROBE-pTetr-AP because it was ampicillin-resistant (80 μg mL^–1^). Kanamycin sensitivity was observed in this strain (50 μg mL^–1^). Biparental mating with donor *Escherichia coli* (*E. coli*) strain TG1 resulted in the introduction of plasmid pPROBE-pTetr-AP (2.6 kb) carrying the GFP and kanamycin genes produced under the control of a Tetr promoter. The recipient and donor strains were combined in a 1:2 ratio. An aliquot (100 μL) of this combination was spread on Luria Bertani (LB) agar and incubated at 30°C for 24 h. After incubation, the bacteria cells were collected and plated onto selective media containing ampicillin (80 mg mL^–1^) and kanamycin (50 mg mL^–1^). A fluorescence microscope was used to select ex-conjugants that fluoresced green under the UV irradiation (Leica Microsystems, Wetzlar, Germany).

### Colonization Analysis Under Gnotobiotic Assays

Active cells of GFP-tagged GXS16 strain was injected into 50 mL of LB medium and incubated at 30°C until late log phase. Centrifugation at 4500 xg for 15 min yielded the tagged bacterial cells. The bacterial cells were then rinsed with phosphate-buffered saline (PBS) at pH 6.5 and the cell density was set to 10^5^ CFU/mL. Micropropagated sugarcane tissue culture plants (variety RB86-7515) were cultivated at 30°C in 50 mL tubes containing 10 mL of agar-free MS media with tagged bacteria. In the control plants, PBS was used in place of the bacterial suspension. After bacterial inoculation, sugarcane tissue culture plantlets (inoculated and uninoculated) were taken out on the first, third, and fifth days and cleaned with autoclaved distilled water. The root tissues were sliced into small pieces and put on a slide with 10% (v/v) glycerol before being examined using a Leica TCS SP5 laser scanning confocal microscope (Leica Microsystems, Mannheim, Germany).

### Nitrogenase Activity Assay

The nitrogenase activity of an isolated strain was determined using gas chromatography (Shimadzu GC-17A) ethylene and the acetylene reduction technique (Baldani et al., 1986). The nitrogenase activity was calculated using the method described by Yao et al. (2004). The experiments were carried out in triplicate.

### Effects of Temperature and pH on Nitrogenase Activity

In a 50 mL flask with a tight-fitting rubber stopper, the strain GXS16 was inoculated into 10 mL sterile culture media (Xing et al., 2016) and incubated at 200 revolution per minute (rpm) for 36 h on rotary shaker. Five mL of acetylene gas was introduced into the flask and incubated at the same rpm for the following 36 h at different temperatures (20, 25, 30, 35, and 40°C, respectively) nitrogenase activity was measured. To determine the effect of pH on nitrogenase activity, culture medium pH was adjusted in the ranges of 6, 7, 8, and 9, using filter-sterilized solutions of sodium hypo-chloride (NaOCl) and hydrochloric acid (HCL), and strain GXS16 was inoculated and followed the 200 rpm on 28°C, and inoculated the acetylene gas, and nitrogenase activity was detected. Both tests were carried out in triplicate.

### Effects of Carbon and Nitrogen Sources on Nitrogenase Activity

Sucrose and glucose were added as carbon sources to the sterile culture medium at various concentrations (1, 5, 10, and 15%). The culture GXS16-inoculated broth flasks were incubated at 28°C at 200 RPM, and nitrogenase activity was measured as described before. In addition, at doses of 0, 2, 4, 6, and 8 g/L, Ammonium sulfate [(NH_4_)_2_SO_4_] and potassium nitrate (KNO_3_) were utilized as nitrogen sources in the growth medium. The culture was incubated at 28°C and 200 rpm for 36 h before being used for the nitrogenase activity test. Both tests were carried out in triplicate.

### Inoculation of Sugarcane Tissue Culture Seedlings With GXS16

Rooting tufted sugarcane tissue culture seedlings were divided into individual plants and placed in a culture flask (1/10 MS liquid media, without vitamins and plant hormones), as Lin et al. (2012) described. The seedlings underwent gnotobiotic culture at 30°C, 14 h light/10 h dark, and 60 μmol photons m^–2^S^–1^ light intensity. After 7 days, the plants with consistent growth were transferred to plastic pots (21 cm in diameter and 19 cm high) with a mixture of thoroughly sterilized sand and perlite [v/v = 1:1], placed in a light incubator for adaptation, and watered with 200 mL 1/10 MS nutrient solution each time as required. After the new roots grew long enough, the GXS16 in the logarithmic phase were collected by centrifugation (30°C, 100 rpm for 5 min), washed twice with 1/10 MS culture solution, and diluted to 2 × 10^8^ CFU mL^–1^ bacterial suspension. A suspension of 200 mL of bacteria was used to water the plant roots. An equal amount of sterile water was used as the control. The young roots of the plants were collected at 1, 3, and 5 days after inoculation. They were packaged in units, quick-frozen in liquid nitrogen, and stored at −80°C.

### Measurement of Physiological Indices

The contents of proline (Pro), malondialdehyde (MDA), and hydrogen peroxide (H_2_O_2_) in the roots were calculated as described by Health and Packer (1968); Bates et al. (1973), and Jaleel et al. (2007), respectively. The activities of catalase (CAT), peroxidase (POD), polyphenol oxidase (PPO), superoxide dismutase (SOD), ascorbate peroxidase (APX), and glutathione reductase (GR) were measured using commercial kits from Suzhou Comin Biotechnology Co., Ltd. (Suzhou, China) following the manufacturer’s instructions.

The contents of abscisic acid (ABA) and gibberellic acid (GA_3_) were measured as described by Iriti et al. (2009). The content of ethylene was measured using gas chromatography (GC) (Shimadzu, Tokyo, Japan). A total of 0.5 g of fresh root sample was placed in a 10 mL wide-mouth bottle with a rubber plug that contained 1 mL of pH 6.8 phosphate buffer to react for 24 h at 30°C in the dark. A volume of 1 mL of gas was extracted from the top of the wide-mouth bottle with a sterile injector. The content of ethylene was measured using Gas Chromatography. Measurement conditions: hydrogen flame ion detector, a temperature of 70°C, a temperature of 150°C in sample introduction chamber, detector temperature of 250°C, air pressure of 49 kPa, H_2_ pressure of 70–80 kPa, and N_2_ pressure of 110 kPa.

### Detection of the Strain Copy Number

The Biospin Omni Plant Genomic DNA Extraction Kit (Bioer Technology, Hangzhou, China) was used to extract the root DNA, and the copy number of GXS16 in the roots was determined using a TaqMan fluorescent probe. The forward and reverse primers were F: 5′-GCAGGCGGTTTGCTAAGACC-3′ and R: 5′-GCTTTCGTGCATGAGCGTCA-3′. The probe sequence was 5′-CGGGCTCAACCTGGGAACTGC-3′. The qRT-PCR reaction condition as: 2 × SYBR Green I Master Mix (TaKaRa, Dalian, China) 10 μL, 0.5 μL each of the 10 μM primers and probe, DNA template 5 μL, and ddH_2_O supplemented to 20 μL. Reaction conditions are as follows: pre-denaturation at 95°C for 30 s; denaturation at 95°C for 5 s, annealing at 60°C for 30 s for 40 cycles. Following the completion of the process, the strain copy number in the roots was determined using a standard curve generated by the GXS16 16S rRNA gene plasmid.

### *De novo* Assembly and Transcriptome Sequencing

BGI Technology Services Co., Ltd. (Shenzhen, China) created the RNA-Seq libraries for the various root samples, which were then sequenced using the BGI SEQ-500 platform. The raw data of the libraries have been uploaded to the National Genomics Data Center with the accession number CRA004951. After quality control, low-quality sequencing reads were filtered out from the raw data, and Bowtie (Langmead and Salzberg, 2012) was used to compare the filtered reads from each sample with the reference database. HTSeq (Anders et al., 2015) was used to calculate gene expression, which is indicated by the fragments per kilobase of transcript per million mapped fragments (FPKM) value. DESeq2 (Love et al., 2014) was used to analyze the gene expression differences between samples. The multiple of difference in expression as |log_2_ Fold Change|≥ 1, and the adjusted *P*-value ≤ 0.05, i.e., the gene expressed a significant difference between samples.

### Gene Function Annotation

BLAST was used to compare the differentially expressed genes (DEGs) with NR, Swiss-Prot, the KEGG (Kyoto Encyclopedia of Genes and Genomes), and NT databases (threshold value is *e*-value < 1e^–5^) for gene function annotation. Blast2GO (Conesa et al., 2005) was used for Gene Ontology (GO) annotation, and WEGO was used for GO classification statistics. The ggplot2 package of R language was used to map the bubble diagram for pathway enrichment analysis.

### Validation of RNAseq Results by Quantitative Real-Time PCR

Sugarcane *GAPDH* (glyceraldehyde-3-phosphate dehydrogenase) was selected as the reference gene. Among the DEGs screened, 10 that were involved in ethylene or ABA metabolic pathways were randomly selected, and specific primers were designed (Supplementary Table 1). The transcriptome RNA from related root samples was extracted and converted into cDNA, and gene expression was confirmed using quantitative real-time PCR (qRT-PCR). The reaction condition is as follows: 10 μL 2 × All-in-One qPCR Mix (Gene Copoeia, Rockville, MD, United States), 2 μL cDNA, and 1 μL each of the 4 μM primers and ddH_2_O up to 20 μL. The reaction proceeds as follows: pre-denaturation at 95°C for 10 min; denaturation at 95°C for 10 s, annealing at 60°C for 20 s, extension at 72°C for 20 s of 40 cycles. Relative expression quantification of the selected DEGs to control *GAPDH* was analyzed using the 2^–ΔΔ*Ct*^ method (Livak and Schmittgen, 2001).

### Data Analysis

Data were analyzed using standard ANOVA followed by the Duncan’s Multiple Range Test (DMRT). The SPSS statistical software was used to accomplish all analyses (SPSS Version 23, SPSS Inc., Chicago, IL, United States). Differences were considered significant at the *p* < 0.05 level. All experiments were carried out in replicate, and the results are given as mean values.

## Results

### Colony Morphology and Colonization of Green Fluorescent Protein Tagged Strain GXS16

**FIGURE 1:**
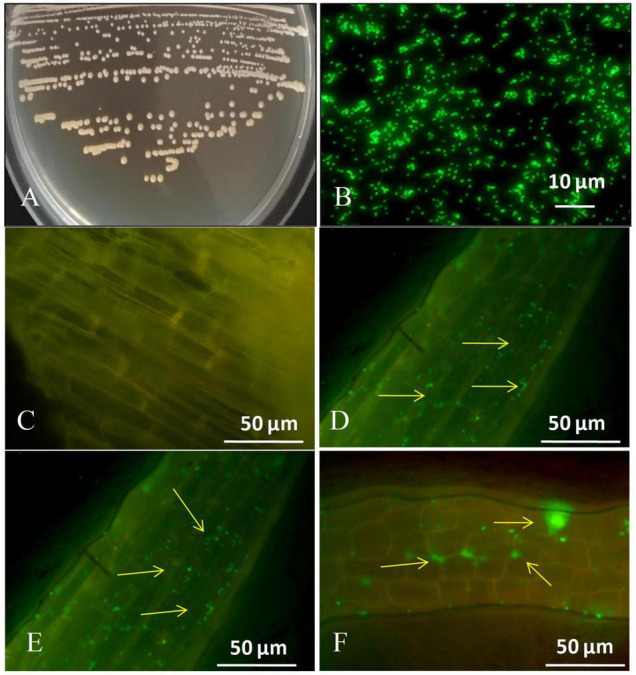
**(A)** Colony appearance of strain GXS16, **(B)** Microscopy of GFP-tagged strain GXS16 cells, **(C)** Non-inoculated plant root (Control), **(D)** GFP tagged strain GXS16 colonized plant root at 1 day after inoculation, **(E)** GFP tagged strain colonized root at 3 days after inoculation, **(F)** GFP tagged strain colonized root at 5 days after inoculation. The presence of bacteria as a single, bunch, or patch is indicated by arrowheads.

Figures 1A,B depicts the colony characteristics as well as the strain’s corresponding GFP-tagged microscopic features. Images taken using a fluorescent confocal microscope indicated that the GFP-labeled strain had infiltrated the roots of sugarcane plantlets (Figure 1). We discovered tagged bacteria penetrate the root surface and elongation zones 1 day after inoculation (Figure 1D). 3 days after inoculation tagged strain GXS16 was attached to roots alone or in clusters (Figure 1E). Furthermore, GFP-tagged bacterial cells were seen as clusters and patches in the root maturation zone on the 5 days after inoculation (Figure 1F). Colonization of GFP-tagged bacteria was readily visible as little specks of green fluorescence.

### Nitrogenase Activity of Strain GXS16

**FIGURE 2:**
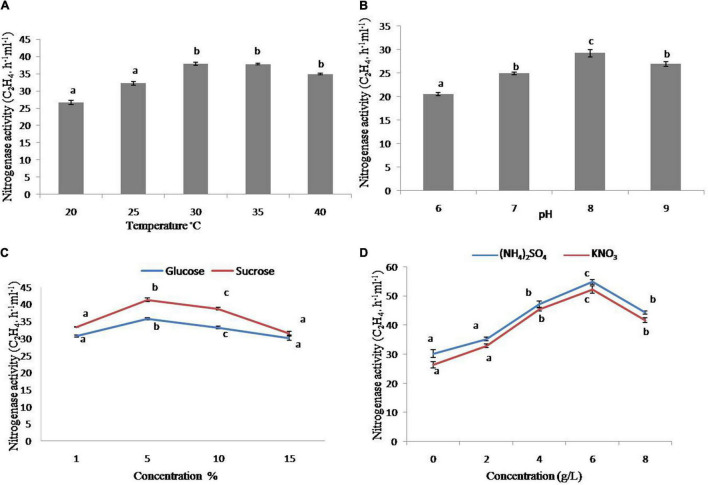
Nitrogenase activity of strain GXS16. **(A)** Temperature, **(B)** pH, **(C)** Glucose and sucrose, **(D)** (NH_4_)_2_SO_4_ and KNO_3_. Values are mean, and vertical bars indicate the standard deviation of three replications. Means followed by the same letter within a column are not significantly different (*p* < 0.05) according to Duncan’s multiple range test (DMRT).

The strain has increased nitrogenase activity in the early testing. As a result, it was employed for further characterization under various physiological and nutritional settings. The nitrogenase activity of the strain was substantially more significant at 30 and 45°C as compared to 20°C, with the maximum at 30°C (Figure 2A), whereas the greatest nitrogenase activity was obtained at pH 8 (Figure 2B). In addition, sucrose and glucose were used as carbon sources. The strain had increased nitrogenase activity with 5% sucrose, whereas glucose had comparable tendencies with various concentrations (1–10%), however, the higher sucrose content had a negative effect on nitrogenase activity (Figure 2C). In terms of nitrogen sources, the maximum nitrogenase activity was seen at 4 and 6 g/L of (NH4)_2_SO_4_ and KNO_3_, respectively (Figure 2D).

### *De novo* Assembly and Transcriptome Sequencing

After assembling all samples together and filtering the abundance, we got 181,345 Unigenes, the total length, average length, N50, and GC content of Unigenes are 243161,718 bp, 1340 bp, 2077 bp and 49.37% respectively. And then annotate Unigenes by aligning with 7 functional databases, finally, 116233 (NR:64.09%), 129265 (NT:71.28%), 72209 (Swissprot:39.82%), 79,321 (KOG:43.74%), 84775 (KEGG:46.75%), 49965 (GO:27.55%), and 74188 (InterPro:40.91%) Unigenes are annotated. Venn diagram show the annotation result of NR, KOG, KEGG, SwissProt, and InterPpro (Supplementary Figure 1).

### Change of Strains Number in Sugarcane Roots

The sugarcane roots at different inoculation times were sampled, and the copy number of GXS16 in the roots was determined using absolute quantitative real-time PCR. copy number calculation formula obtained from the 16S rRNA gene plasmid reaction result was as follows: Y = −3.406x + 37.05 [y: *Ct* value; x: Log_10_ (copy number)]. As shown in Table 1, the number of GXS16 strains in the roots rapidly increased with the extension of inoculation time.

**TABLE 1:** The copy number of GXS16 strain in sugarcane roots.

Treatment	Average *Ct* value	*X*-value	Copies (0.05 g^–1^)
Control-1	32.09	1.46	2.86 × 10^2^
Inoculation-1	26.95	2.96	9.23 × 10^3^
Control-3	31.26	1.69	5.01 × 10^2^
Inoculation-3	17.21	5.82	6.68 × 10^6^
Control-5	32.13	1.44	2.75 × 10^2^
Inoculation-5	15.31	6.38	2.41 × 10^7^

### Changes in Physiological Parameter in Sugarcane Roots

**FIGURE 3:**
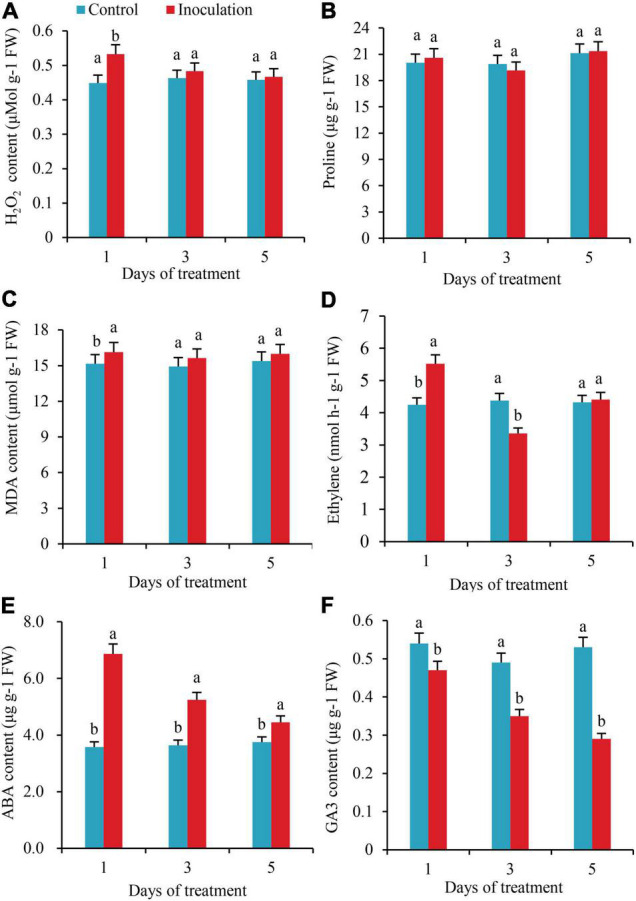
Effect of inoculation with GXS16 on **(A)** H_2_O_2_, **(B)** Proline, **(C)** MDA, **(D)** Ethylene, **(E)** ABA, and **(F)** GA_3_. Values are mean, and vertical bars indicate the standard deviation of three replications. Means followed by the same letter within a column are not significantly different (*p* < 0.05) according to Duncan’s multiple range test (DMRT). H_2_O_2_, hydrogen peroxide; MDA, malondialdehyde; ABA, abscisic acid; GA_3_, gibberellic acid.

On the first day after inoculation, the contents of H_2_O_2_ in the roots increased by 15.8% as compared to the control, with the difference reaching a significant level; however, on the third and fifth days after inoculation, the contents of H_2_O_2_ in the roots increased by 4.1 and 1.9%, respectively, compared with the control, with no significant difference (Figure 3A). The whole treatment period, there was no significant variation in the content of Pro in the roots (Figure 3B). On the first day after inoculation, the content of MDA in the roots increased by 6.4% compared with the control, but on the third and fifth days after inoculation, the content of MDA did not change significantly from the control (Figure 3C). On the first day after inoculation, the ethylene content in roots increased by 29.9%, while on the third day, it decreased by 23.3%, with significant differences. In addition, on the fifth day after inoculation, the ethylene contents in the two treatments did not differ significantly (Figure 3D). On the first, third, and fifth days after inoculation, the contents of ABA increased by 91.9, 43.9, and 18.7% compared with those of the control, respectively, with significant differences (Figure 3E). In contrast to the changing trend of ABA, the contents of GA_3_ decreased by 12.9, 28.5, and 45.2% compared with the control, respectively, with significant differences (Figure 3F).

### Changes of Antioxidant Enzymes Activities in Sugarcane Roots

**FIGURE 4:**
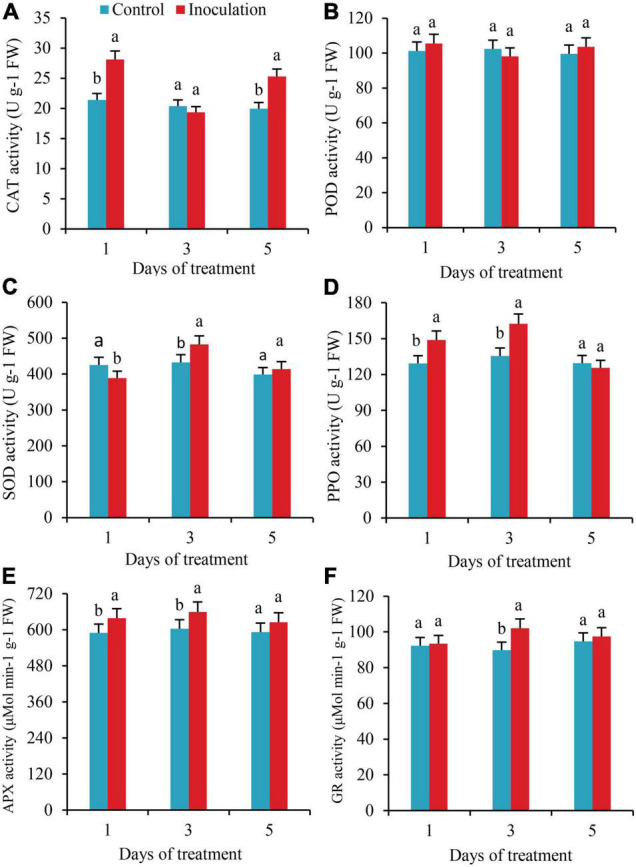
Effect of Inoculation with GXS16 on **(A)** CAT, **(B)** POD, **(C)** SOD, **(D)** PPO, **(E)** AXP, and **(F)** GR activity in sugarcane roots. Values are mean, and vertical bars indicate the standard deviation of three replications. Means followed by the same letter within a column are not significantly different (*p* < 0.05) according to Duncan’s multiple range test (DMRT). CAT, catalase; POD, peroxidase; PPO, polyphenol oxidase; SOD, superoxide dismutase; PPO, polyphenol oxidase; AXP, ascorbate peroxidase; GR, glutathione reductase.

The activities of CAT significantly increased by 31.3 and 26.6%, respectively, on the first and fifth days after inoculation. Still, there was no significant difference in CAT activity between the two treatments on the third day (Figure 4A). There were no substantial changes detected in POD activity within 5 days of inoculation (Figure 4B). On the first day after inoculation, the SOD activity of the roots was significantly lower than that of the control. On the third day after inoculation, the SOD activity increased by 11.6%, with significant differences (Figure 4C). The activities of PPO and APX were significantly higher than those of the control on the first and third days after inoculation, increasing by 15.2 and 19.9% and 8.4 and 9.9%, respectively, but on the fifth day after inoculation, there was no significant difference in these activities compared with the control (Figures 4D,E). On the third day after inoculation, the activity of GR increased by 13.7%, with a significant difference (Figure 4F).

### Identification of Differentially Expressed Genes

**FIGURE 5:**
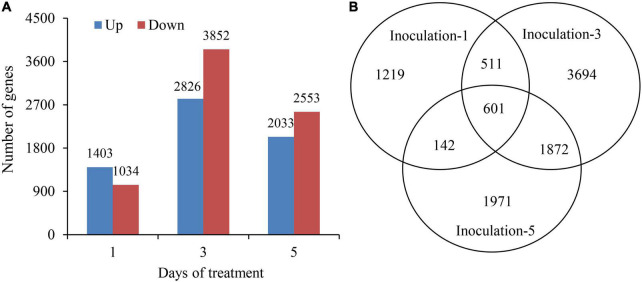
Summary of differentially expressed genes at 1st, 3rd, and 5th days after inoculation. **(A)** Up and down regulated differentially expressed genes. **(B)** Venn diagram showing the differentially expressed gene at all three sampling conditions.

The DEseq2 algorithm was used to detect DEGs in the samples at each inoculation period compared with the control. The total numbers of DEGs on the first, third, and fifth days after inoculation were 2,437, 6,678, and 4,586, respectively, and the highest number of DEGs was on the third day after inoculation. The numbers of up and downregulated DEGs in different sampling days were 1,403 and 1,034; 2,826 and 3,852; and 2,033 and 2,553, respectively. Additionally, the DEGs were preferentially induced in the early inoculation stage (first day), and the downregulated expression of genes played a dominant role in the latter stage (fifth day) (Figure 5A). Notably, there were 601 genes significantly expressed overlapped in all three sampling conditions (Figure 5B).

### Gene Ontology Classification of Differentially Expressed Genes

**FIGURE 6:**
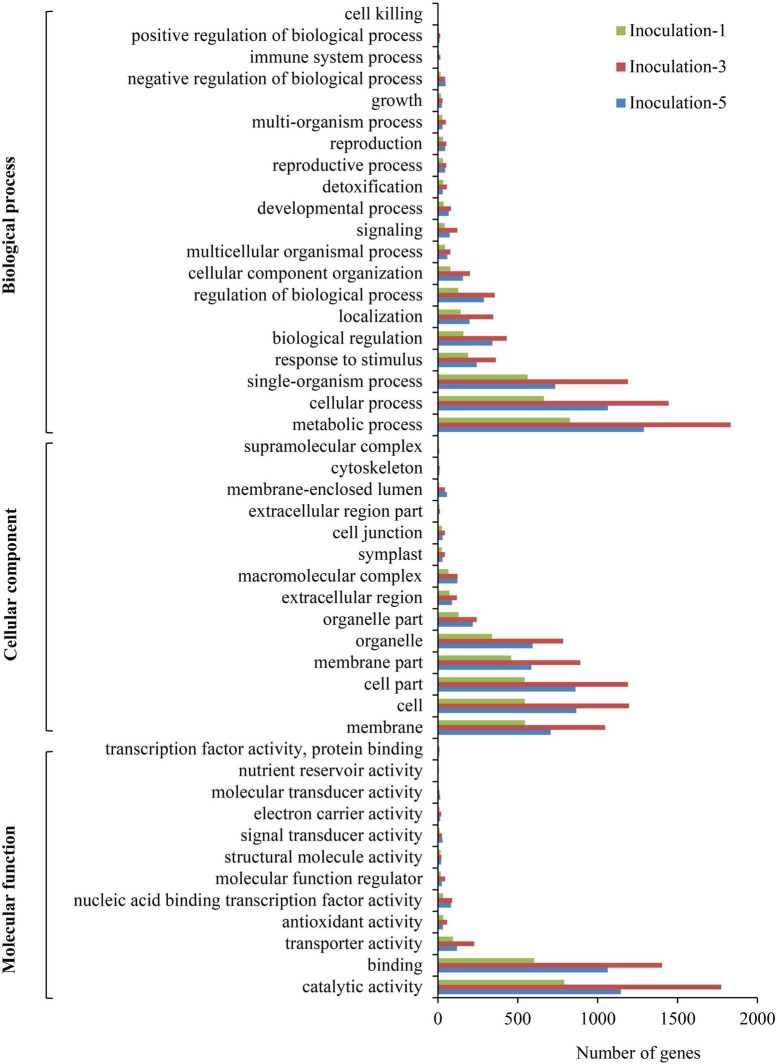
Pathway functional enrichment of differentially expressed genes. Inoculation-1, -3, and -5 represent inoculation treatments on the 1st, 3rd, and 5th day, respectively.

The GO annotations for DEGs were submitted to the Web Gene Ontology Annotation Plot (WEGO) for GO classification. In summary, the DEGs were assigned to 46 GO categories (Figure 6). Among them, 20, 14, and 12 categories belonged to biological processes, cellular components, and molecular functions, respectively. In the biological process category, the metabolic process, cellular process, and single-organism process comprised the top three terms with respect to the number of DEGs. For cellular components, the cell, cell part, and membrane part categories were the top three with respect to the number of genes. The DEGs were mainly involved in catalytic activity, binding, transporter activity, antioxidant activity, and nucleic acid binding transcription factor activity.

### Pathway Classification of Differentially Expressed Genes

**FIGURE 7:**
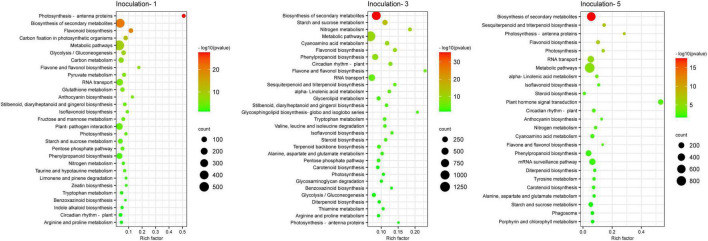
Gene Ontology classification of differential expression genes.

A KEGG database comparison showed that the DEGs could be annotated into five level-1 or 19 level-2 metabolic pathways. In the specific metabolic pathways, on the first, third, and fifth days after inoculation, a total of 1,932, 4,852, and 3,400 DEGs were annotated into 120, 131, and 127 metabolic pathways, respectively (Supplementary Table 2), and there were 29, 31 and 25 metabolic pathways with DEGs that were significantly enriched (*P* ≤ 0.05), respectively, in each sampling days (Figure 7), among which 12 metabolic pathways were improved considerably in each inoculation period, including the biosynthesis of secondary metabolites, circadian rhythm-plant, flavone and flavonol biosynthesis, flavonoid biosynthesis, isoflavonoid biosynthesis, nitrogen metabolism, metabolic pathways, phenylpropanoid biosynthesis, photosynthesis, photosynthesis-antenna proteins, rRNA transport, and starch and sucrose metabolism.

### Plant Hormone Related Genes Analysis

**FIGURE 8:**
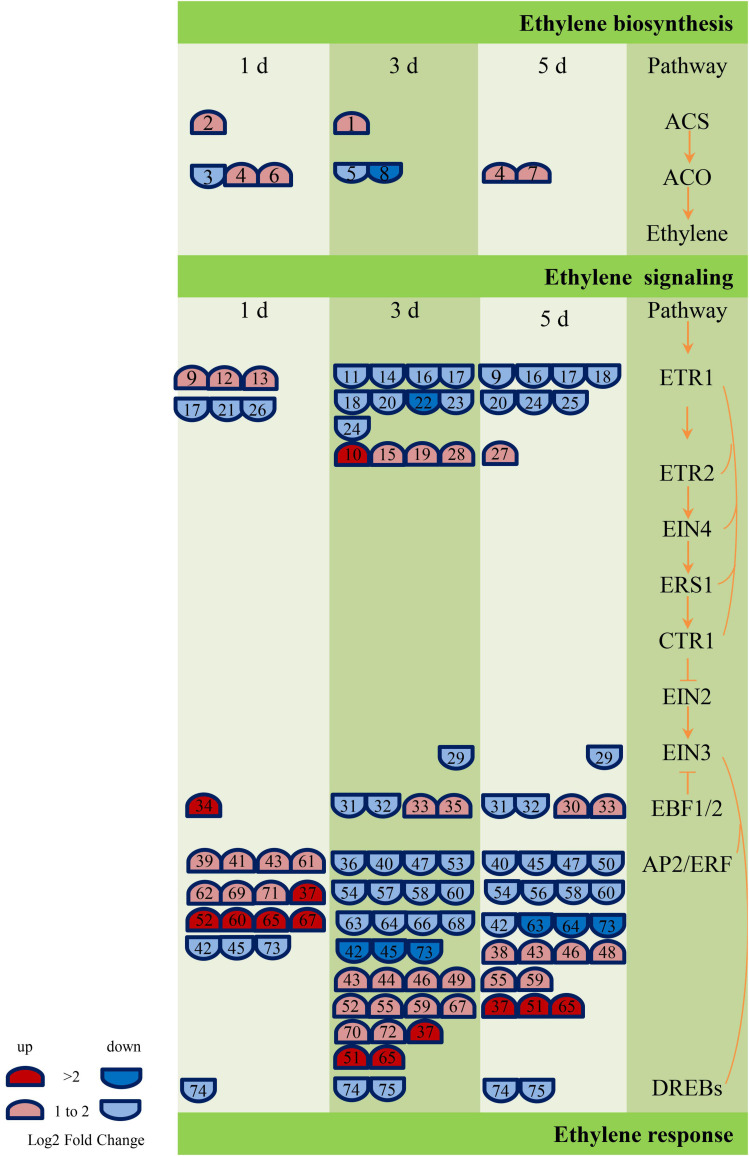
Ethylene biosynthesis and signaling-related genes responses to GXS16 colonization in sugarcane roots. Gene expression levels (log2 fold change) are represented as colors and symbols. Red stands for upregulated and blue stands for downregulated genes. The DEGs are identified with numbers which are illustrated in Supplementary Table 5.

According to the annotation results of GO and KEGG, the genes related to ABA, GA, and ethylene metabolic pathway were screened further, and 17 (Supplementary Table 3), 42 (Supplementary Table 4), and 75 (Supplementary Table 5) DEGs were obtained, respectively. These genes are engaged in biochemical activities such as biological processes of biosynthesis, metabolism, transport, or signal transduction of these plant hormones. However, the number of genes related to ethylene metabolism was much higher than GA and ABA. In addition, most of the genes related to the ABA metabolic pathway (NCED, PP2C, and SNRK2) were downregulated, and most of the GA signal transduction gene DELLA was upregulated, while the other signal transduction genes (GID1 and PIF) were primarily downregulated. The largest number of DEGs (38) encoded the ethylene signaling gene AP2/ERF (Ethylene-responsive transcription factor). The AP2/ERF genes were predominantly upregulated on the first day of inoculation (12 genes were upregulated, and three genes were downregulated), and the number of upregulated and those of the downregulated genes were equal on the third and fifth days of inoculation. Figure 8 and Supplementary Table 5 show the details of the number and expression fold changes of the ethylene signal transduction genes.

### Validation of RNA-Seq Data by Quantitative Real-Time PCR

**FIGURE 9:**
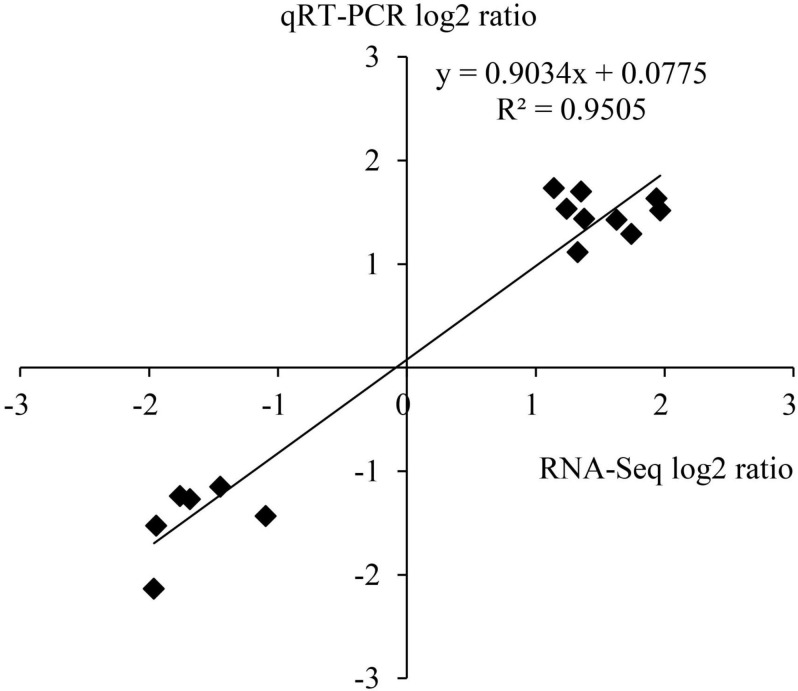
Correlation between RNA-seq and qRT-PCR.

To verify the results of transcriptome sequencing analysis, 10 DEGs occupied in the ethylene and ABA metabolic pathways (Table 1) were randomly selected for qRT-PCR detection. The correlation coefficient *R*^2^ of data on the change of gene expression obtained by the two methods reached 0.95 (Figure 9), which indicates that the transcriptome sequencing data in this study are reliable and repeatable.

## Discussion

In the traditional methods of cultivating sugarcane in China, the excessive application of nitrogen fertilizer poses a severe problem (Yang et al., 2014) that leads to high costs of sugarcane production and further reduces the international competitiveness of its sugarcane industry. Furthermore, due to the low rate of nitrogen use in sugarcane production (about 14.5–24.7%), a considerable portion of the nitrogen is volatilized, leached, and absorbed by the soil. Resulting in the loss of a large amount of nitrogen, which causes severe waste and becomes an essential non-point source pollution factor after entering the environment (Li and Yang, 2015). Making full use of sugarcane’s biological nitrogen fixation characteristics and reducing the amount of nitrogen fertilizer applied are effective ways to minimize fertilization and increase benefits. It is also highly significant in promoting the sustainable development of the sugarcane industry in China (Li and Yang, 2015). The roots have been shown to have an essential role in the biological nitrogen fixation of sugarcane. Compared with other tissues and organs, the roots provide better carbohydrates for the function of nitrogenase, and the partial pressure of oxygen in the roots and their surroundings is lower, which can effectively prevent the oxidation of nitrogenase. Therefore, it contributes to a higher percentage of nitrogen fixation (Hu et al., 2017). Close interaction between the NFB and sugarcane roots is the basis for NFB to fix nitrogen effectively. The present study discovered that GFP-tagged bacteria successfully colonize the sugarcane root and bacteria enter root maturation zone at fifth day. The number of GFP-tagged bacteria rose as the inoculation period increased, as seen by microscopic characteristics, and this is confirmed by quantitative real-time PCR. The quantity of gene copies rose as the inoculation time increased. Wei et al.,2014 used fluorescence microscopy to colonize a GFP-tagged *Klebsiella* strain in the interior of the roots and aerial portions of micropropagated sugarcane plantlets. *Burkholderia anthina* MYSP13 strain was also found in lateral root emergence locations indicating that colonization occurred in sugarcane root and shoot (Malviya et al., 2019). Recently endophytic plant growth promoting strain *Enterobacter roggenkampii* ED5 labeled with GFP had been effectively colonized the sugarcane tissues (Guo et al., 2021).

In this study, we established that GXS16 has great nitrogen fixation capability in a somewhat alkaline atmosphere and strong nitrogenase activity at temperatures of 40°C, allowing it easier to survive and play a role in nitrogen fixation in sugarcane tropical and subtropical environment. According to Luna et al. (2000), the carbon supply is sometimes more significant than the nitrogen source for *Acetobacter diazotrophicus* strains with nitrogen fixation capabilities. In this investigation, sucrose outperformed glucose for the investigated strain GXS16. Xing et al. (2016) discovered that The nitrogenase activities of *Stenotrophomonas maltophili* and *Agrobacterium tumefaciens* rose with rising concentrations of NH4^+^ and NO3^–^ in a limited range, but when the nitrogen concentration exceeded the critical values, the nitrogenase activity of the two strains declined. This is consistent with the current study’s findings. Actinobacteria strain also showed similar nitrogenase activity at varying temperatures, pH levels, and carbon and nitrogen sources, according to Wang et al. (2016).

The RNA-seq approach was employed in this work to investigate the molecular basis of interaction between sugarcane cultivars in response to an N-fixing endophytic strain inoculation, to observe the physiological and hormonal changes in sugarcane plant roots. The defense response plays a vital role in the perception of bacterial invasion by plants. When foreign bacteria invade a plant, even if the phenotype of the plant has no noticeable change, the internal tissues and cells of the plant will undergo programmed death owing to the invasion of the bacteria, and a series of ROS and similar compounds are generated to kill the invading microorganisms (Alqueres et al., 2013; Zhou et al., 2015). As signal molecules, a low concentration of ROS regulates the expression of genes in cells. In contrast, a high concentration of ROS will cause severe damage to cell membranes and proteins, with lipid peroxidation of the cell membranes as the most typical case. The content of MDA indicates the severity of injury (Singh et al., 2016). By using sugarcane variety RB86-7515 as tissue culture seedlings, this study examined the effects of inoculation of the dominant endogenous NFB GXS16 on the physiological and biochemical products and expression of genes of the sugarcane roots. H_2_O_2_ and MDA varied consistently in the roots. They were significantly higher than the control in the early stage of inoculation. Still, They showed no significant change during the middle and later stages, which indicates that GXS16 only triggers the root defense response in the early stage of inoculation and has no significant effect in the middle and later stages. It also confirms the results of previous studies (Vandenkoornhuyse et al., 2015), which have shown that the response of plants to stress can initiate the protective enzymatic clearance system (Zhang et al., 2019), the non-enzymatic clearance system (Zhan et al., 2021), and other approaches to ensure the normal function of their own cells. The clearance of active oxygen in plants is completed collaboratively by the protective system of antioxidant enzymes and compounds. In this study, the activities of CAT, SOD, PPO, APX, and GR in the roots increased significantly following inoculation with GXS16, which could be the reason why the contents of ROS and MDA in the middle and late stages (third and fifth days after inoculation) did not differ significantly from those in the control. In addition, ROS at low concentrations can be used as signal molecules to regulate the expression of genes in root cells, which is conducive to the colonization of roots by nitrogen-fixing strains. The change in the copy number of GXS16 strains in roots shows that the number of cells in colonized roots increases with the extension of inoculation time. It has also been demonstrated that proline, as an osmoregulatory substance, also affects the production and clearance of ROS (Gerona et al., 2019). In this study, there was no significant difference in proline content between the roots inoculated with GXS16 and the control, indicating that antioxidant enzymes also play a dominant role in the elimination of ROS.

Plant hormones play important role in the resistance response to endophytic bacterial colonization (Ibort et al., 2018). In this study, the content of ethylene in the roots increased first and then decreased after inoculation with GXS16. Although the increment gradually reduced, the content of ABA was significantly higher than that of the control during the whole treatment period, and the trend in the change of GA_3_ was opposite to that of ABA. Previous studies have shown that ABA-dependent metabolic pathways play vital role in the response of plants to abiotic stress (Li et al., 2015, 2019). The content of endogenous ABA in plants under stress increased rapidly. In this study, 17 DEGs related to ABA metabolism were obtained by transcriptomic sequencing, including the NCED gene, which is associated with ABA biosynthesis, and the PP2C and SnRK2 genes that are related to ABA signal transduction. Under normal conditions, PP2C combines with SnRK2 to dephosphorylate SnRK2 and deactivate it (Chen et al., 2020). ABA combines with a receptor protein and then acts on PP2C to change its conformation and inhibit its enzyme activity. The activity of SnRK2 negatively regulated by PP2C can be released to activate the expression of target genes in response to ABA signals and control the reaction of plants to stress conditions (Chen et al., 2020). However, whether sugarcane roots respond to the colonization of NFB GXS16 through this pathway remains to be clarified.

Different plant hormones can exert their biological functions synergistically or antagonistically through complex and cross-connected network-like regulatory pathways to regulate the process of plant development (Berens et al., 2017). As a pair of classical hormone combinations, GA and ABA have antagonistic biological effects on seed dormancy, taproot development, seedling growth, and stress responses (Lin et al., 2015; Shu et al., 2018). This could be why the trend in variation of GA_3_ content in this study was opposite to that of ABA. Studies indicate that ethylene biosynthesis and its signal transduction components play an essential role in the adaptation of plants to colonization by bacterial endophytes (Ibort et al., 2018; Nascimento et al., 2018). In this study, there were 75 DEGs involved in ethylene biosynthesis (*ACS* and *ACO*) or signal transduction (CTR1, ENI3, EBF1/2, AP2/ERF, and DREB). The diversity of both gene number and expression is much higher than that of DEGs related to the metabolism of ABA and GA, indicating that ethylene could play a more important role in the response of sugarcane roots to GXS16 colonization. When the plants are subjected to stress, ethylene will be produced, and high levels of ethylene can inhibit the elongation of plant roots and promote the aging and abscission of plant organs (Pan et al., 2019; Singh et al., 2021). 1-Aminocyclopropane-1-carboxylic acid (ACC) is a direct precursor of ethylene biosynthesis (Pattyn et al., 2021). Studies have shown that under adverse conditions, some bacteria that have ACC deaminase activity and colonize plants can absorb the ACC secreted by plant cells and catalyze the ACC deamination reaction through ACC deaminase to generate α-ketobutyric acid and NH_3_ as carbon and nitrogen sources for bacterial growth (Barnawal et al., 2017; Liu et al., 2021), respectively, reducing the concentration of ACC in plant cells and reducing the production of ethylene, which alleviates the inhibition of accumulation of ethylene on plant growth. Our previous study showed that the NFB GXS16 have high ACC deaminase activity (Nong et al., 2021). However, more research is needed to clarify whether it is involved in the regulation of plant hormones in the response of roots to colonization by GXS16 and whether it is correlated with the number of DEGs and the diversity of gene expression.

## Conclusion

The plant hormones GA, ABA, and ethylene all play important roles in sugarcane roots’ response to GXS16 colonization. GXS16 inoculation increases the amount of H_2_O_2_, MDA, and ABA in sugarcane roots while decreasing the concentration of GA3. The ethylene concentration rises at first, then falls. The activity of antioxidant enzymes in the roots increases dramatically, which effectively clears the formation of excess ROS and lowers the buildup of MDA-induced membrane lipid peroxidation products.

## Data Availability Statement

The datasets presented in this study can be found in online repositories. The names of the repository/repositories and accession number(s) can be found in the article/Supplementary Material.

## Author Contributions

CL and Y-RL initiated and designed the research. QN, MM, LL, JX, ZM, ZW, X-PS, and XH performed the laboratory experiments together. QN, CL, MM, and MS wrote the manuscript. MS, AS, SR, and Y-RL revised the manuscript. All authors reviewed the manuscript and approved it for publication.

## Conflict of Interest

The authors declare that the research was conducted in the absence of any commercial or financial relationships that could be construed as a potential conflict of interest.

## Publisher’s Note

All claims expressed in this article are solely those of the authors and do not necessarily represent those of their affiliated organizations, or those of the publisher, the editors and the reviewers. Any product that may be evaluated in this article, or claim that may be made by its manufacturer, is not guaranteed or endorsed by the publisher.
