# Supplementary material for: Sugarcane Root Transcriptome Analysis Revealed the Role of Plant Hormones in the Colonization of an Endophytic Diazotroph
Source: Front Microbiol. 2022 Jun 24;13:924283. doi: 10.3389/fmicb.2022.924283 (PMC9263702; doi:10.3389/fmicb.2022.924283)
Supplement: Supplementary file 1 [file Data_Sheet_1.PDF]

Supplementary figure

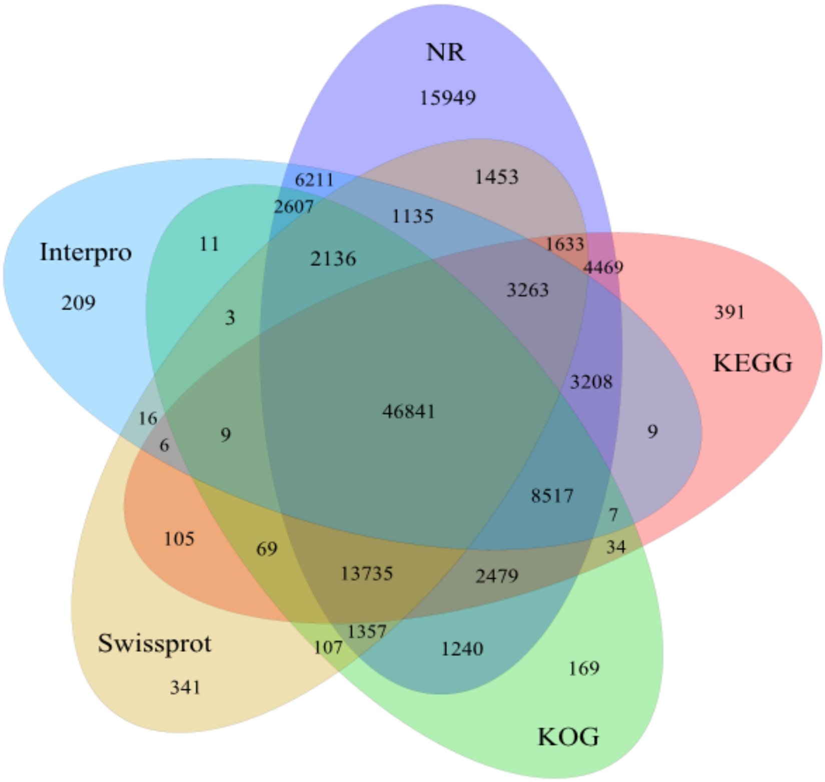

Figure S1. Venn diagram between NR, KOG, KEGG, Swissprot and Interpro

Supplementary tables:

Table S1 Genes and primers list for qRT-PCR

Gene No.	Gene name	Forward primer (5'-3')	Reverse primer (5'-3')
EF189713	GAPDH	TGGTGCTGACTATGTCGTGGA	CATGGGTGCATCTTTGCTTG
CL10478	ACS6	AAAACGGGCACACCCTACAG	GGTCCACGCCAGCTAGTCA
CL15902	ACO	GCAATCTATCTACTCGATCGTCTTCA	GAAGTCGATCACGGGAACCA
Unigene57805	EIN3	CGAGCGGGATGGAGAAGTAG	TCTGGAGCCTGGACAACATCT
CL14864	ETR1	GGTGTTACTGGCTCTCCTTTTCAA	CCAATGCAAGCAGCAAGGT
CL18927	ERF12	TGATCCATGATCCCTACTCGATACT	CGATCTCCATGAACGCACAT
CL8770	NCED	TGCTGGACAAGGAGAAGACG	AGGTGGAAGCAGAAGCAGTC
CL4677	SnRK2	CCACCATACCACCTGCCTGTTCT	ACAATCTCACCGCTGCTGTCAA
CL1290	PP2C	CTGTCCGACCAGATCAGAAAC	CCAGTTCACATTCGCTTCG
CL8131	DREB1A	TGGCGGCTCCACGAGTAG	TGGACTTGGAATTGGATGTGTACTA
Unigene19520	PYL8	CCGAGGGCCAGGTCATCA	TATATCTCCCATCACCGGTCAAG

Table S2 Pathway classifications of differential expression genes

	Inoculation-1			Inoculation-3			Inoculation-5		
Pathway	DEGs number	% of annotated DEGs	p_value	DEGs number	% of annotated DEGs	p_value	DEGs number	% of annotated DEGs	p_value
Photosynthesis - antenna proteins	27	1.40	2.5E-28	8	0.16	4.3E-02	15	0.44	6.8E-08
Biosynthesis of secondary metabolites	408	21.12	3.1E-21	948	19.54	3.1E-36	632	18.59	3.2E-18
Flavonoid biosynthesis	53	2.74	2.1E-20	64	1.32	9.5E-10	47	1.38	1.6E-07
Carbon fixation in photosynthetic organisms	50	2.59	3.3E-12	43	0.89	4.5E-01	17	0.50	1.0E+00
Metabolic pathways	549	28.42	5.2E-10	1267	26.11	1.1E-10	865	25.44	1.8E-05
Glycolysis / Gluconeogenesis	53	2.74	2.4E-09	67	1.38	3.3E-02	44	1.29	2.5E-01
Carbon metabolism	90	4.66	9.5E-08	140	2.89	7.3E-02	80	2.35	1.0E+00
Flavone and flavonol biosynthesis	13	0.67	1.9E-07	17	0.35	1.1E-05	10	0.29	5.8E-03
Pyruvate metabolism	32	1.66	1.6E-06	27	0.56	1.0E+00	16	0.47	1.0E+00
RNA transport	180	9.32	2.8E-06	388	8.00	2.4E-05	289	8.50	7.2E-06
Glutathione metabolism	42	2.17	5.0E-06	53	1.09	3.5E-01	29	0.85	1.0E+00
Anthocyanin biosynthesis	12	0.62	1.6E-05	8	0.16	4.5E-01	12	0.35	3.8E-03
Stilbenoid, diarylheptanoid and gingerol biosynthe	21	1.09	4.7E-05	34	0.70	8.0E-04	20	0.59	7.8E-02
Isoflavonoid biosynthesis	15	0.78	6.5E-05	22	0.45	1.8E-03	18	0.53	2.0E-03
Fructose and mannose metabolism	26	1.35	8.2E-05	27	0.56	7.6E-01	18	0.53	1.0E+00
Plant-pathogen interaction	172	8.90	1.8E-04	317	6.53	9.5E-01	235	6.91	5.1E-01
Photosynthesis	15	0.78	2.2E-04	20	0.41	3.0E-02	26	0.76	1.1E-06
Pentose phosphate pathway	26	1.35	3.6E-04	44	0.91	9.0E-03	16	0.47	1.0E+00
Starch and sucrose metabolism	64	3.31	3.6E-04	186	3.83	4.9E-16	90	2.65	1.9E-02
Phenylpropanoid biosynthesis	118	6.11	1.6E-03	306	6.31	3.6E-09	188	5.53	6.4E-03
Nitrogen metabolism	16	0.83	2.5E-03	49	1.01	1.6E-11	23	0.68	4.1E-03
Taurine and hypotaurine metabolism	12	0.62	3.1E-03	15	0.31	2.0E-01	12	0.35	1.8E-01
Limonene and pinene degradation	9	0.47	5.7E-03	9	0.19	4.8E-01	4	0.12	1.0E+00
Zeatin biosynthesis	8	0.41	8.6E-03	5	0.10	1.0E+00	5	0.15	8.9E-01
Tryptophan metabolism	16	0.83	2.4E-02	37	0.76	9.8E-04	15	0.44	9.3E-01
Benzoxazinoid biosynthesis	7	0.36	2.4E-02	12	0.25	3.1E-02	7	0.21	2.7E-01
Indole alkaloid biosynthesis	13	0.67	2.7E-02	21	0.43	2.0E-01	9	0.26	1.0E+00
Circadian rhythm - plant	21	1.09	4.5E-02	67	1.38	1.8E-08	39	1.15	2.3E-03
Arginine and proline metabolism	14	0.72	4.5E-02	28	0.58	4.1E-02	14	0.41	8.7E-01
Fatty acid elongation	10	0.52	5.6E-02	11	0.23	1.0E+00	10	0.29	6.8E-01
Amino sugar and nucleotide sugar metabolism	56	2.90	8.4E-02	119	2.45	3.2E-01	67	1.97	1.0E+00

Continiue

Pathway	Inoculation-1			Inoculation-3			Inoculation-5		
	DEGs number	% of annotated DEGs	p_value	DEGs number	% of annotated DEGs	p_value	DEGs number	% of annotated DEGs	p_value
Thiamine metabolism	9	0.47	8.4E-02	19	0.39	3.5E-02	10	0.29	5.3E-01
Vitamin B6 metabolism	5	0.26	8.4E-02	9	0.19	7.3E-02	5	0.15	4.7E-01
Biosynthesis of amino acids	60	3.11	8.6E-02	119	2.45	8.1E-01	61	1.79	1.0E+00
Porphyrin and chlorophyll metabolism	18	0.93	1.1E-01	36	0.74	2.1E-01	32	0.94	2.2E-02
Phagosome	19	0.98	2.3E-01	44	0.91	1.1E-01	37	1.09	1.9E-02
Tropane, piperidine and pyridine alkaloid biosynth	8	0.41	2.3E-01	12	0.25	8.4E-01	7	0.21	1.0E+00
Cyanoamino acid metabolism	26	1.35	2.7E-01	100	2.06	3.3E-10	55	1.62	4.1E-03
Lysine biosynthesis	6	0.31	3.0E-01	10	0.21	6.0E-01	3	0.09	1.0E+00
beta-Alanine metabolism	9	0.47	3.5E-01	19	0.39	3.6E-01	10	0.29	1.0E+00
Glycerolipid metabolism	23	1.19	3.5E-01	71	1.46	6.7E-04	43	1.26	9.5E-02
mRNA surveillance pathway	124	6.42	3.8E-01	317	6.53	5.8E-02	241	7.09	6.4E-03
Cutin, suberine and wax biosynthesis	9	0.47	4.0E-01	21	0.43	2.3E-01	16	0.47	2.5E-01
Sulfur relay system	4	0.21	4.1E-01	3	0.06	1.0E+00	7	0.21	2.7E-01
Alanine, aspartate and glutamate metabolism	10	0.52	4.1E-01	31	0.64	8.1E-03	23	0.68	1.6E-02
Histidine metabolism	4	0.21	4.1E-01	7	0.14	6.0E-01	1	0.03	1.0E+00
Folate biosynthesis	6	0.31	4.9E-01	12	0.25	6.0E-01	7	0.21	1.0E+00
Monobactam biosynthesis	5	0.26	5.3E-01	10	0.21	6.0E-01	3	0.09	1.0E+00
Biosynthesis of unsaturated fatty acids	4	0.21	5.6E-01	12	0.25	7.4E-02	5	0.15	1.0E+00
Glycerophospholipid metabolism	24	1.24	5.6E-01	42	0.87	1.0E+00	38	1.12	9.3E-01
alpha-Linolenic acid metabolism	9	0.47	6.3E-01	38	0.78	8.1E-05	29	0.85	3.4E-04
Sulfur metabolism	6	0.31	6.5E-01	11	0.23	1.0E+00	14	0.41	1.1E-01
Glucosinolate biosynthesis	1	0.05	6.6E-01	2	0.04	5.0E-01			
Other types of O-glycan biosynthesis	6	0.31	6.8E-01	9	0.19	1.0E+00	5	0.15	1.0E+00
RNA degradation	27	1.40	7.2E-01	43	0.89	1.0E+00	43	1.26	1.0E+00
Ribosome	29	1.50	7.2E-01	62	1.28	1.0E+00	42	1.24	1.0E+00
Protein export	12	0.62	7.4E-01	35	0.72	1.7E-01	27	0.79	1.4E-01
Glycine, serine and threonine metabolism	15	0.78	7.6E-01	33	0.68	1.0E+00	31	0.91	2.8E-01
Phenylalanine metabolism	9	0.47	7.6E-01	23	0.47	5.6E-01	15	0.44	9.5E-01
Phosphonate and phosphinate metabolism	2	0.10	7.6E-01	1	0.02	1.0E+00			
Cysteine and methionine metabolism	18	0.93	8.1E-01	38	0.78	1.0E+00	28	0.82	1.0E+00
Monoterpenoid biosynthesis	2	0.10	8.2E-01	6	0.12	4.1E-01	3	0.09	1.0E+00
Citrate cycle (TCA cycle)	11	0.57	8.7E-01	11	0.23	1.0E+00	9	0.26	1.0E+00

Continiue

	Inoculation-1			Inoculation-3			Inoculation-5		
Pathway	DEGs number	% of annotated DEGs	p_value	DEGs number	% of annotated DEGs	p_value	DEGs number	% of annotated DEGs	p_value
Carotenoid biosynthesis	10	0.52	8.7E-01	37	0.76	2.8E-02	30	0.88	8.5E-03
Aminoacyl-tRNA biosynthesis	16	0.83	9.3E-01	34	0.70	1.0E+00	21	0.62	1.0E+00
Diterpenoid biosynthesis	7	0.36	9.4E-01	28	0.58	3.4E-02	24	0.71	7.1E-03
One carbon pool by folate	2	0.10	9.4E-01		0.00		1	0.03	1.0E+00
Ether lipid metabolism	7	0.36	9.4E-01	14	0.29	1.0E+00	10	0.29	1.0E+00
Sesquiterpenoid and triterpenoid biosynthesis	5	0.26	9.4E-01	30	0.62	6.8E-05	31	0.91	4.7E-08
2-Oxocarboxylic acid metabolism	16	0.83	1.0E+00	35	0.72	1.0E+00	19	0.56	1.0E+00
Valine, leucine and isoleucine degradation	6	0.31	1.0E+00	33	0.68	1.8E-03	17	0.50	3.0E-01
Terpenoid backbone biosynthesis	11	0.57	1.0E+00	52	1.07	2.9E-03	29	0.85	3.0E-01
Inositol phosphate metabolism	9	0.47	1.0E+00	26	0.54	1.0E+00	17	0.50	1.0E+00
Lysine degradation	7	0.36	1.0E+00	21	0.43	1.0E+00	4	0.12	1.0E+00
MAPK signaling pathway - plant	52	2.69	1.0E+00	145	2.99	1.0E+00	105	3.09	1.0E+00
Steroid biosynthesis	4	0.21	1.0E+00	30	0.62	2.2E-03	24	0.71	2.3E-03
Fatty acid degradation	7	0.36	1.0E+00	23	0.47	1.0E+00	21	0.62	6.6E-01
Glyoxylate and dicarboxylate metabolism	12	0.62	1.0E+00	48	0.99	4.5E-01	35	1.03	4.7E-01
Tyrosine metabolism	5	0.26	1.0E+00	21	0.43	9.4E-01	27	0.79	8.4E-03
Glycosaminoglycan degradation	3	0.16	1.0E+00	24	0.49	3.0E-02	14	0.41	3.2E-01
Proteasome	3	0.16	1.0E+00	6	0.12	1.0E+00	4	0.12	1.0E+00
Non-homologous end-joining	1	0.05	1.0E+00	2	0.04	1.0E+00	1	0.03	1.0E+00
Sphingolipid metabolism	5	0.26	1.0E+00	32	0.66	5.3E-02			
Arginine biosynthesis	7	0.36	1.0E+00	28	0.58	9.6E-01	15	0.44	1.0E+00
Base excision repair	7	0.36	1.0E+00	17	0.35	1.0E+00	14	0.41	1.0E+00
Pentose and glucuronate interconversions	17	0.88	1.0E+00	56	1.15	1.0E+00	31	0.91	1.0E+00
Phosphatidylinositol signaling system	9	0.47	1.0E+00	30	0.62	1.0E+00	17	0.50	1.0E+00
Ascorbate and aldarate metabolism	12	0.62	1.0E+00	35	0.72	1.0E+00	21	0.62	1.0E+00
Isoquinoline alkaloid biosynthesis	2	0.10	1.0E+00	8	0.16	1.0E+00	8	0.24	1.0E+00
Pantothenate and CoA biosynthesis	2	0.10	1.0E+00	5	0.10	1.0E+00	3	0.09	1.0E+00
Glycosylphosphatidylinositol (GPI)-anchor biosynt	3	0.16	1.0E+00	8	0.16	1.0E+00	6	0.18	1.0E+00
SNARE interactions in vesicular transport	1	0.05	1.0E+00	1	0.02	1.0E+00			
Oxidative phosphorylation	8	0.41	1.0E+00	20	0.41	1.0E+00	19	0.56	1.0E+00
Arachidonic acid metabolism	2	0.10	1.0E+00	8	0.16	1.0E+00	6	0.18	1.0E+00
Propanoate metabolism	1	0.05	1.0E+00	2	0.04	1.0E+00	4	0.12	1.0E+00

Continiue

	Inoculation-1			Inoculation-3			Inoculation-5		
Pathway	DEGs number	% of annotated DEGs	p_value	DEGs number	% of annotated DEGs	p_value	DEGs number	% of annotated DEGs	p_value
Galactose metabolism	13	0.67	1.0E+00	60	1.24	4.8E-01	27	0.79	1.0E+00
Nicotinate and nicotinamide metabolism	5	0.26	1.0E+00	14	0.29	1.0E+00	6	0.18	1.0E+00
Butanoate metabolism	1	0.05	1.0E+00	12	0.25	9.0E-01	6	0.18	1.0E+00
Ubiquinone and other terpenoid-quinone biosynthe	4	0.21	1.0E+00	24	0.49	1.0E+00	20	0.59	8.5E-01
Phenylalanine, tyrosine and tryptophan biosynthesi	2	0.10	1.0E+00	15	0.31	1.0E+00	3	0.09	1.0E+00
Plant hormone signal transduction	42	2.17	1.0E+00	143	2.95	1.0E+00	141	4.15	2.3E-03
Fatty acid metabolism	5	0.26	1.0E+00	21	0.43	1.0E+00	11	0.32	1.0E+00
Ribosome biogenesis in eukaryotes	7	0.36	1.0E+00	15	0.31	1.0E+00	9	0.26	1.0E+00
RNA polymerase	19	0.98	1.0E+00	52	1.07	1.0E+00	31	0.91	1.0E+00
Selenocompound metabolism	4	0.21	1.0E+00	10	0.21	1.0E+00	7	0.21	1.0E+00
Purine metabolism	35	1.81	1.0E+00	99	2.04	1.0E+00	65	1.91	1.0E+00
Homologous recombination	10	0.52	1.0E+00	30	0.62	1.0E+00	22	0.65	1.0E+00
Basal transcription factors	3	0.16	1.0E+00	13	0.27	1.0E+00	10	0.29	1.0E+00
ABC transporters	10	0.52	1.0E+00	33	0.68	1.0E+00	38	1.12	1.0E+00
Fatty acid biosynthesis	2	0.10	1.0E+00	12	0.25	1.0E+00	9	0.26	1.0E+00
Peroxisome	8	0.41	1.0E+00	51	1.05	8.4E-01	36	1.06	9.6E-01
Spliceosome	25	1.29	1.0E+00	85	1.75	1.0E+00	51	1.50	1.0E+00
Ubiquitin mediated proteolysis	12	0.62	1.0E+00	34	0.70	1.0E+00	20	0.59	1.0E+00
Mismatch repair	7	0.36	1.0E+00	33	0.68	1.0E+00	20	0.59	1.0E+00
DNA replication	8	0.41	1.0E+00	30	0.62	1.0E+00	25	0.74	1.0E+00
Other glycan degradation	6	0.31	1.0E+00	38	0.78	1.0E+00	27	0.79	1.0E+00
Protein processing in endoplasmic reticulum	32	1.66	1.0E+00	112	2.31	1.0E+00	78	2.29	1.0E+00
Endocytosis	35	1.81	1.0E+00	145	2.99	1.0E+00	87	2.56	1.0E+00
Nucleotide excision repair	5	0.26	1.0E+00	29	0.60	1.0E+00	20	0.59	1.0E+00
Pyrimidine metabolism	19	0.98	1.0E+00	67	1.38	1.0E+00	41	1.21	1.0E+00
Betalain biosynthesis				1	0.02	5.0E-01			
Biotin metabolism				2	0.04	1.0E+00	2	0.06	1.0E+00
Brassinosteroid biosynthesis				2	0.04	1.0E+00	2	0.06	1.0E+00
Caffeine metabolism				1	0.02	1.0E+00	1	0.03	1.0E+00
Glycosphingolipid biosynthesis - ganglio series				13	0.27	4.1E-01	11	0.32	2.6E-01
Glycosphingolipid biosynthesis-globo and isoglobo series				12	0.25	8.0E-04	5	0.15	2.8E-01
Linoleic acid metabolism				7	0.14	1.0E+00	2	0.06	1.0E+00

Continiue

Pathway	Inoculation-1			Inoculation-3			Inoculation-5		
	DEGs number	% of annotated DEGs	p_value	DEGs number	% of annotated DEGs	p_value	DEGs number	% of annotated DEGs	p_value
Mannose type O-glycan biosynthesis				1	0.02	1.0E+00			
N-Glycan biosynthesis				7	0.14	1.0E+00	7	0.21	1.0E+00
Riboflavin metabolism				8	0.16	9.7E-01	2	0.06	1.0E+00
Synthesis and degradation of ketone bodies				8	0.16	5.1E-02	2	0.06	1.0E+00
Valine, leucine and isoleucine biosynthesis				3	0.06	1.0E+00	1	0.03	1.0E+00

Note: Inoculation-1, -3, -5 represent inoculation treatment at 1st, 3rd and 5th day, respectively. DEGs number, % of annotated DEGs and p_value indicated the number of DEGs which annotated to specific pathway, the percentage to total annotated DEGs and the calculation significance of those genes, respectively.

Table S3 Filtering results of differential expression genes related to ABA biosynthesis and signal transduction pathway

Gene name	Gene abbr.	Gene ID	Annotated species	e_value	Fold Change		
					1 d	3 d	5 d
9-cis-epoxycarotenoid dioxygenase	NCED	CL8770	Zay mays	0.00E+00	—	1.522	1.938
		CL9591	Arabidopsis thaliana	1.30E-20	—	-1.885	-2.009
		Unigene57218	Zay mays	0.00E+00	-2.215	—	—
		Unigene57219	Zay mays	1.70E-286	—	1.428	—
Protein phosphatase 2C	PP2C	CL1290	Arabidopsis thaliana	4.40E-115	—	—	-1.349
		CL141	Oryza sativa	2.20E-33	—	-1.227	-1.3
		CL16599	Oryza sativa	3.20E-57	—	—	1.184
		CL16717	Arabidopsis thaliana	1.20E-34	—	-1.245	—
		CL18262	Oryza sativa	3.90E-13	—	1.059	—
		CL2552	Arabidopsis thaliana	1.30E-16	—	-1.017	-1.248
		CL2825	Zay mays	5.50E-70	—	1.21	—
		CL498	Oryza sativa	4.90E-123	—	-1.741	—
		CL6298	Arabidopsis thaliana	5.60E-31	1.101	—	-1.017
		Unigene10989	Oryza sativa	4.60E-10	—	—	-1.262
		Unigene49846	Oryza sativa	7.80E-16	—	—	1.167
		Unigene60972	Zay mays	1.40E-169	—	1.063	1.24
Serine/threonine-protein kinase SRK	SnRK2	CL4677	Oryza sativa	5.90E-144	—	1.74	—

—: Indicated non-significant difference of gene expression between control and inoculation at the same time.

Table S4 Filtering results of differential expression genes related to gibberellin biosynthesis and signal transduction pathway

Gene name	Gene abbr.	Gene ID	Annotated species	e_value	Fold Change		
					1 d	3 d	5 d
Gibberellin receptor	GID1	Unigene947	Zay mays	3.46E-04	-1.555	—	—
DELLA protein	DELLA	Unigene18083	Arabidopsis thaliana	7.03E-04	—	2.353	—
		Unigene25367	Zay mays	4.01E-05	—	2.262	—
		Unigene8925	Zay mays	3.60E-04	—	1.404	—
		Unigene21782	Arabidopsis thaliana	2.12E-09	—	1.360	—
		Unigene61761	Oryza sativa	2.62E-03	—	1.157	1.396
		Unigene11329	Oryza sativa	2.72E-03	—	1.213	—
		CL20339	Arabidopsis thaliana	1.05E-05	—	1.178	—
		Unigene29641	Oryza sativa	1.63E-03	—	1.091	—
		CL12728	Arabidopsis thaliana	2.17E-03	—	1.077	—
		Unigene26959	Zay mays	1.09E-03	—	1.040	—
		CL4410	Oryza sativa	1.15E-05	—	1.095	—
		CL89	Arabidopsis thaliana	6.44E-04	—	-1.978	—
		CL6048	Oryza sativa	1.18E-03	—	—	1.672
		CL8065	Oryza sativa	4.69E-04	—	—	1.057
Phytochrome-interacting factor	PIF	CL4653	Zay mays	7.76E-04	1.460	—	-1.099
		CL14743	Zay mays	1.11E-04	-1.649	—	—
		CL423	Oryza sativa	5.20E-05	-1.557	—	-1.905
		CL7795	Arabidopsis thaliana	1.21E-05	-1.531	-1.366	-1.337
		CL10804	Zay mays	1.96E-06	-1.769	-1.918	—
		CL15581	Oryza sativa	1.49E-05	—	-1.064	—
		CL1607	Arabidopsis thaliana	1.30E-03	—	1.795	—
		CL18276	Oryza sativa	2.08E-05	—	2.879	—
		CL19871	Oryza sativa	2.14E-03	—	-1.108	-1.698
		CL2726	Zay mays	3.37E-08	—	-1.394	-1.712
		CL6429	Zay mays	4.08E-05	—	-1.224	—
		CL6747	Zay mays	7.46E-07	—	1.723	—
		Unigene27783	Oryza sativa	3.90E-04	—	-1.052	-1.249
		Unigene28294	Arabidopsis thaliana	4.17E-09	—	-1.255	-1.138
		Unigene3738	Zay mays	3.21E-15	—	-1.669	-1.431
		Unigene39334	Oryza sativa	2.90E-04	—	-2.202	—
		Unigene4202	Arabidopsis thaliana	2.35E-05	—	-1.252	-1.283
		Unigene4204	Oryza sativa	1.56E-17	—	-1.846	-1.430
		Unigene57907	Oryza sativa	2.24E-19	—	-1.835	-1.446
		Unigene64997	Zay mays	7.06E-23	—	-1.537	-1.358
		Unigene66115	Arabidopsis thaliana	5.03E-06	—	-1.505	-1.061
		CL19459	Arabidopsis thaliana	1.62E-05	—	—	1.355
		CL19471	Arabidopsis thaliana	1.23E-04	—	—	-2.351
		CL2203	Zay mays	9.74E-04	—	—	-1.490
		Unigene49528	Arabidopsis thaliana	1.29E-05	—	—	1.760
		Unigene49529	Arabidopsis thaliana	2.62E-06	—	—	1.260
		Unigene49530	Arabidopsis thaliana	1.52E-04	—	—	1.336

—: Indicated non-significant difference of gene expression between control and inoculation at the same time.

Table S5 Filtering results of differential expression genes related to ethylene biosynthesis and signal transduction pathway

Gene name	Gene abbr.	Gene No.	Gene ID	Annotated species	e_value	Fold Change		
						1 d	3 d	5 d
1-aminocyclopropane-1-carboxylate synthase 1	ACS	1	CL10478	Oryza sativa	3.90E-252	—	1.141	—
		2	CL4949	Oryza sativa	5.20E-173	1.161	—	—
1-aminocyclopropane-1-carboxylate oxidase	ACO	3	CL11800	Arabidopsis thaliana	2.50E-18	-1.295	—	—
		4	CL15902	Arabidopsis thaliana	1.10E-85	1.239	—	1.03
		5	CL18911	Arabidopsis thaliana	2.10E-24	—	-1.506	—
		6	CL49	Arabidopsis thaliana	4.70E-92	1.281	—	—
		7	Unigene16188	Arabidopsis thaliana	4.20E-159	—	—	1.011
		8	Unigene46282	Arabidopsis thaliana	8E-87	—	-2.511	—
Serine/threonine-protein kinase CT	CTR1	9	CL6298	Arabidopsis thaliana	2.5E-55	1.101	—	-1.017
		10	CL6374	Arabidopsis thaliana	4.10E-43	—	2.206	—
		11	CL7469	Arabidopsis thaliana	1.90E-41	—	-1.266	—
		12	CL14873	Arabidopsis thaliana	5.80E-39	1.578	—	—
		13	CL14978	Arabidopsis thaliana	5.60E-45	1.57	—	—
		14	CL16717	Arabidopsis thaliana	9.90E-56	—	-1.373	—
		15	CL18262	Arabidopsis thaliana	1.50E-20	—	1.059	—
		16	CL141	Arabidopsis thaliana	2.00E-50	—	-1.227	-1.3
		17	CL173	Arabidopsis thaliana	2.10E-15	-1.69	-1.241	-1.051
		18	CL20270	Arabidopsis thaliana	8.10E-22	—	-1.268	-1.469
		19	CL2531	Arabidopsis thaliana	1.60E-46	—	1.308	—
		20	CL2552	Arabidopsis thaliana	1.00E-32	—	-1.158	-1.323
		21	CL4523	Arabidopsis thaliana	3.00E-86	-1.639	—	—
		22	CL5267	Arabidopsis thaliana	1.40E-49	—	-2.476	—
		23	CL5517	Arabidopsis thaliana	3.40E-156	—	-1.167	—
		24	CL173	Arabidopsis thaliana	1.60E-15	—	-1.404	-1.191
		25	Unigene10989	Arabidopsis thaliana	2.20E-12	—	—	-1.262
		26	Unigene41641	Arabidopsis thaliana	2.20E-44	-1.26	—	—
		27	Unigene49846	Arabidopsis thaliana	3.60E-21	—	—	1.167
		28	Unigene62223	Arabidopsis thaliana	4.10E-48	—	1.116	—
Ethylene-insensitive protein 3	ENI3	29	Unigene57805	Arabidopsis thaliana	3.90E-11	—	-1.953	-1.684
EIN3-binding F-box protein	EBF1/2	30	CL7057	Arabidopsis thaliana	1.60E-09	—	—	1.535
		31	CL7695	Arabidopsis thaliana	2.90E-08	—	-1.237	-1.417
		32	CL7912	Arabidopsis thaliana	2.00E-13	—	-1.391	-1.358
		33	CL8195	Arabidopsis thaliana	2.30E-14	—	1.693	2.337
		34	CL2773	Arabidopsis thaliana	6.20E-20	2.159	—	—
		35	Unigene42230	Arabidopsis thaliana	5.30E-42	—	1.094	—
Ethylene-responsive transcription factor	AP2/ERF	36	CL10278	Arabidopsis thaliana	4.60E-48	—	-1.002	—
		37	CL1041	Arabidopsis thaliana	1.80E-27	3.079	2.611	2.953
		38	CL10677	Arabidopsis thaliana	2.90E-39	—	—	1.639
		39	CL11153	Arabidopsis thaliana	5.60E-25	1.803	—	—
		40	CL113	Oryza sativa	1.10E-134	—	-1.205	-1.184
		41	CL14183	Arabidopsis thaliana	6.20E-33	1.07	—	—
		42	CL1731	Arabidopsis thaliana	1.30E-19	-1.189	-2.324	-1.865
		43	CL18927	Arabidopsis thaliana	1.40E-43	1.377	1.965	1.353
		44	CL3406	Arabidopsis thaliana	1.70E-16	—	1.238	—
		45	CL422	Arabidopsis thaliana	7.30E-26	-1.326	-2.057	-1.699
		46	CL4764	Arabidopsis thaliana	5.70E-31	—	1.212	1.273
		47	CL5979	Arabidopsis thaliana	2.30E-25	—	-1.461	-1.354
		48	CL6104	Arabidopsis thaliana	8.20E-27	—	—	1.521
		49	CL7581	Arabidopsis thaliana	2.20E-28	—	—	-1.069
		50	CL6386	Arabidopsis thaliana	1.70E-34	—	1.029	—

Continue

Gene name	Gene abbr.	Gene No.	Gene ID	Annotated species	e_value	Fold Change		
						1 d	3 d	5 d
		51	CL8103	Arabidopsis thaliana	1.80E-10	2.975	2.266	2.586
		52	Unigene10597	Arabidopsis thaliana	1.30E-43	—	1.627	—
		53	Unigene11724	Oryza sativa	1.60E-137	—	-1.055	—
		54	Unigene11799	Arabidopsis thaliana	1.40E-22	—	-1.445	-1.576
		55	Unigene11880	Arabidopsis thaliana	4.90E-38	—	1.593	1.383
		56	Unigene12817	Oryza sativa	5.70E-138	—	—	-1.034
		57	Unigene12959	Arabidopsis thaliana	1.90E-13	—	-1.712	—
		58	Unigene15076	Arabidopsis thaliana	3.70E-25	—	-1.443	-1.279
		59	Unigene16178	Arabidopsis thaliana	1.90E-26	2.573	1.573	1.944
		60	Unigene16235	Arabidopsis thaliana	1.20E-21	—	-1.22	-1.078
		61	Unigene16401	Arabidopsis thaliana	2.30E-31	1.378	—	—
		62	Unigene20462	Arabidopsis thaliana	4.70E-32	1.589	—	—
		63	Unigene21551	Arabidopsis thaliana	5.20E-25	—	-1.652	-2.41
		64	Unigene27666	Arabidopsis thaliana	4.40E-26	—	-1.927	-2.578
		65	Unigene27959	Arabidopsis thaliana	3.90E-28	3.367	2.167	2.469
		66	Unigene31002	Arabidopsis thaliana	1.20E-23	—	-1.384	—
		67	Unigene31123	Arabidopsis thaliana	1.60E-33	2.038	1.859	—
		68	Unigene34659	Arabidopsis thaliana	9.40E-26	—	-1.099	—
		69	Unigene52932	Arabidopsis thaliana	3.10E-39	1.107	—	—
		70	Unigene53860	Arabidopsis thaliana	3.10E-16	—	1.481	—
		71	Unigene6762	Arabidopsis thaliana	1.10E-33	1.07	—	—
		72	Unigene7623	Arabidopsis thaliana	6.80E-38	—	1.424	—
		73	Unigene9977	Arabidopsis thaliana	1.10E-36	-1.031	-2.301	-2.367
Dehydration-responsive element-binding protein	DREB	74	CL8131	Oryza sativa	5.30E-81	-1.159	-1.645	-1.095
		75	Unigene1382	Oryza sativa	5.00E-31	—	-1.537	-1.353

—: Indicated non-significant difference of gene expression between control and inoculation at the same time.
